# CASAS: Cancer Survival Analysis Suite, a web based application

**DOI:** 10.12688/f1000research.11830.2

**Published:** 2017-07-31

**Authors:** Manali Rupji, Xinyan Zhang, Jeanne Kowalski

**Affiliations:** 1 Winship Cancer Institute of Emory University, Atlanta, GA, 30322, USA; 2 Department of Biostatistics and Bioinformatics, Rollins School of Public Health, Emory University, Atlanta, GA, 30322, USA

**Keywords:** survival, quantile survival, landmark, competing risk

## Abstract

We present CASAS, a shiny R based tool for interactive survival analysis and visualization of results. The tool provides a web-based one stop shop to perform the following types of survival analysis:  quantile, landmark and competing risks, in addition to standard survival analysis.  The interface makes it easy to perform such survival analyses and obtain results using the interactive Kaplan-Meier and cumulative incidence plots.  Univariate analysis can be performed on one or several user specified variable(s) simultaneously, the results of which are displayed in a single table that includes log rank p-values and hazard ratios along with their significance. For several quantile survival analyses from multiple cancer types, a single summary grid is constructed. The CASAS package has been implemented in R and is available via http://shinygispa.winship.emory.edu/CASAS/. The developmental repository is available at https://github.com/manalirupji/CASAS/.

## Introduction

Kaplan-Meier (KM) estimates and the Cox Proportional Hazards model have gained huge popularity among clinicians when depicting survival trends and in identifying prognostic biomarkers in cancer research. There is a range of commercial software (SAS, STATA, SPSS, PRISM) available for researchers to carry out survival analysis. However, these programs have several disadvantages; commercial software is proprietary and involves restricted usage with rigid outputs, which cannot be changed easily. Open source software such as R is gaining popularity, but the user needs to learn programming skills, which may be very time consuming for clinicians and biomedical researchers with limited programming exposure.

Standard survival analysis involves a single cause of failure. However, in other cases, clinicians may encounter many other causes of failure in addition to a specific cause of interest. In such cases, a competing risk analysis needs to be carried out, where an individual is exposed to two or more causes of failure but its eventual failure is due to only one cause. While several packages are available to conduct competing risk survival analysis in R, making the right choice presents another layer of confusion to the user.

As opposed to traditional KM or Cox regression analysis, typically a risk factor measured at baseline is examined for its association with survival thereafter. During follow-up, however, things may have changes, such that either the effect of a fixed baseline risk factor may vary over time, resulting in a weakening or strengthening of associations over time or the risk factor itself may vary over time. In the former case, such as effect is often seen in what appears to be significant differences in survival, not necessarily overall and among all survival times, but early on or at later survival times. We address such time-dependent effects on survival by creating two additional tools, one for landmark ^1^ and another for quantile survival analysis ^2, 3^. As an example, the user may want to study the effect of chemotherapy on a specific cancer population and thus divides the data into a responder vs a non-responder group. The issue with this approach is that the responder cannot be deemed one, unless they survive until the time of response. In addition, being in the responder group gives them an unfair survival advantage leading to an immortal time bias. To overcome these issues, the investigator may need to perform a landmark analysis by removing the patients with an event (or censored) before the landmark time from the analysis.

Most tools are available as separate packages. As an alternative, CASAS provides a comprehensive survival analysis suite of tools commonly encountered in cancer research. By providing a GUI interface, the user can readily perform any number of these analyses by simply uploading their data and selecting the variables relevant to the analysis.

In summary, CASAS suite of tools is a one-stop shop for conducting some of the most common survival analyses in cancer research without requiring any prior programming knowledge. It is a web-based application that, as a single tool, can carry out KM plot, univariate hazard ratio, landmark analysis, quantile survival analysis and competing risk analysis. It allows a user to combine results from various studies or cancer types as well.

## Methods

### KM survival analysis

**Figure 1.**
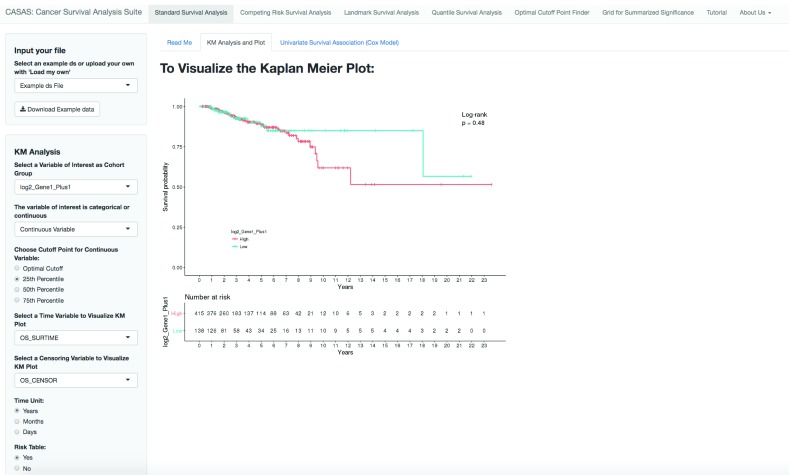
Kaplan-Meier (KM) survival analysis plot. The interface shows an example KM plot using TCGA BRCA data with patients who received radiotherapy ^4^. The left side comprises a user menu and the right includes the result plots/tables. The KM plot is interactive and will change depending on the categorical variable selected. Continuous data can be divided using 25 ^th^ percentile, 50 ^th^ percentile, and 75 ^th^ percentile or the user may opt to use an optimal cut point based on martingale residuals using the ‘survMisc’ package (also available with plot in a separate tab). The user could alternatively choose the “All patients” drop down. If the user selects the ‘All patients’ dropdown, a single KM curve is created using all the data instead of two separate KM curves by the categorical variable of interest. User could also choose to output the number of patients at risk. Univariate survival association analysis based on cox proportional hazards model, can be output by entering one variable in the model each time. Multiple variables can be selected to generate the output table. In addition, the user can also test for the proportional hazard assumption by selecting “Test for Proportional Hazards Assumption” as “Yes”. An additional column with the p-value for Proportional Hazards Assumption will be displayed as the rightmost column.

Standard survival analysis uses the TCGA BRCA data for 553 patients that underwent radiotherapy ^4^ and uses the ‘survminer’ package to display the KM plots. Either categorical or continuous variables can be used for stratification. Continuous variables can be dichomotimized by either the 25 ^th^, 50 ^th^, 75 ^th^ percentile or an optimal cut point. A log-rank test is used to estimate the overall differences between the survival curves ( Figure 1). A single overall survival curve without stratification can be plotted using the ‘All patients’ option. The user can also test for validity of the proportional hazards assumption (a non-significant Schoenfeld residual test p-value based on the cox.zph function in the ‘survival’ package) in the univariate survival analysis tab.

### Competing risk survival analysis

**Figure 2.**
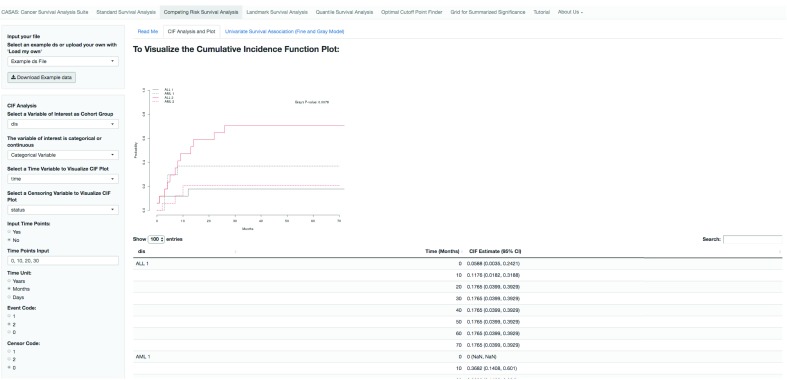
Competing risk analysis. This tab includes competing risk analysis using the BMT data ( http://www.stat.unipg.it/luca/R/). The left panel includes options to select variables for univariate survival association analysis based on Fine and Gray’s model, and includes options to select variables for cumulative incidence function analysis. For the Univariate Analysis, the user chooses variable of interest to enter into the analysis as well as the event and the censor code. To generate a Cumulative Incidence Function (CIF) plot and table, the user can choose either a categorical variable to compare or ‘All Patients’ (Like in Figure 1). Users can also select the appropriate time unit based on the data and the time points of interest, where applicable. Censor code can also be specified. The univariate result table or plot is displayed on the right panel.

Competing risk survival analysis is based on Fine and Gray’s Model ^5^, using the ‘cmprsk’ package in R. To illustrate, 35 AML/ALL patient’s data who underwent Hematopoietic stem cell transplantation (HSCT) affected by either AML or ALL ^6^ are used. Cumulative incidence plot was plotted using CumIncidence.R function available in the package. This tool also displays the Gray’s p-value based on the competing risk code ( Figure 2). Either categorical or continuous group variables/‘All patients’ can be used.

### Landmark analysis

**Figure 3.**
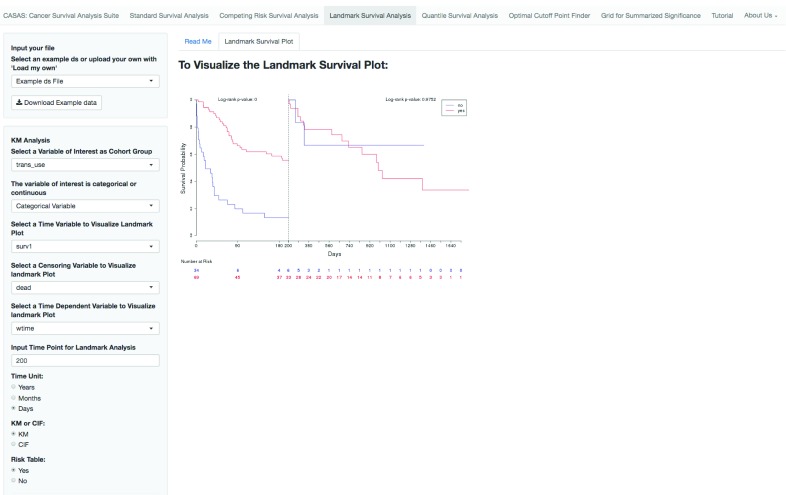
Landmark analysis. This tab includes landmark analysis using the Stanford heart transplant data ^7^. The left panel includes options to select variables for landmark analysis and the landmark time. The program generates a new landmark dataset to create either KM/CIF plots on the right panel. Users can also select the appropriate time unit based on the data.

Landmark analysis is based on a user input landmark time. Stanford Heart transplant dataset is used ^7^. The tool generates an overall KM plot and a landmark KM plot with log rank test p-values ( Figure 3). The user can also opt for a CI curve instead of a KM plot and allows similar categorical/continuous variable inputs.

### Quantile survival analysis

**Figure 4.**
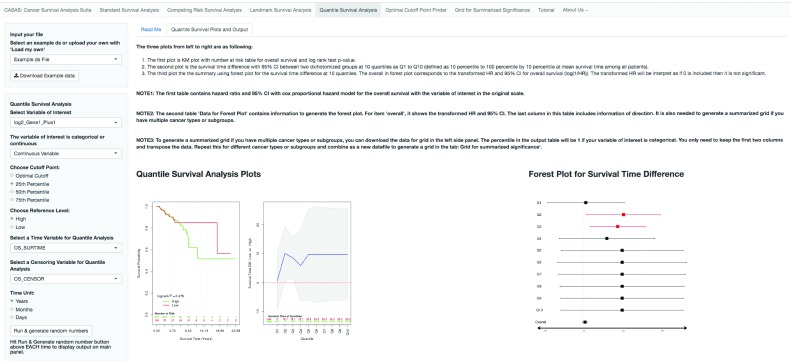
Quantile survival analysis. The quantile regression tab shows three plots based on the same data used in Figure 1. The first is the overall KM plot with number at risk table for overall survival and log rank test p-value. The second is the survival time difference with 95% CI between two dichotomized groups at the 10 quantiles Q1 to Q10 (defined as 10 percentile to 100 percentile by 10 percentile at mean survival time among all patients). The third plot is the summary using forest plot for the survival time difference at 10 quantiles. The overall in forest plot corresponds to the transformed HR and 95% CI for overall survival (log [1/HR]).

Quantile survival analysis is based on method developed by 2, 3, implemented in the ‘cequre’ package in R. The example data used is the 553 patient Expression with Clinical data for TCGA – BRCA patients given radiotherapy ^4^. Survival time difference between the dichotomized continuous or categorical variable will be estimated with 95% CI for each quantile ( Figure 4). A forest plot to represent quantile wise differences between the means and the overall differences is also provided as output.

## Implementation

The CASAS software is written in R and tested using version 3.3.0. The Interactive KM, CIF plots and data tables are made visible through a web browser using the shiny R package ( www.rstudio.com/shiny).

## Operation

Using a windows 7 Enterprise SP1 PC with a 32.0 GB RAM and an 3.30 GHz Intel ^®^ Xeon ^®^ Processor E5 Family, for 553 TCGA BRCA patients who received radiotherapy ^4^, it took 2.26 s to create an interactive KM plot and 6.52 s to generate quantile analysis plots. Stanford heart transplant data ^7^ for landmark survival analysis generated the KM plots in about 1.10s. For data of 35 patients who underwent Hematopoietic stem cell transplantation (HSCT) affected by either AML or ALL ^6^, it took 2.66s to display the CIF plot. The developmental repository is available at https://github.com/manalirupji/CASAS/.

Archived source code as at the time of publication is available at: http://doi.org/10.5281/zenodo.832845 ^8^.

## Discussion

CASAS is a suite of tools that allows a user to conduct various types of survival analysis through an interactive application in R. We show by example, various types of cancer survival analyses that can be performed based on the questions of interest. Our tool will serve as a platform for many physicians and researchers to conduct preliminary analyses before heading to statisticians to conduct advanced analyses.

## Data and software availability

The CASAS web tool ( http://shinygispa.winship.emory.edu/CASAS/) includes preprocessed example data under each tab. The user could use the example data or could upload a dataset of their choice in the same format as the example data. The tool will work best with data input as a .txt or .csv file. Time variable can be input in days, months or years and the appropriate time unit selection can be made. This tool accepts censor variables in various formats, for example, character annotation such as Dead/Alive, or integer variable 0/1, etc. The tool may throw errors if there are missing data within the censor variables but it can handle missing data left blank for the cohort variables. Use of any other characters for missing data may cause the program to throw errors.

For landmark analysis, data from 103 patients on the waitlist for Stanford Heart transplant program as available in the survival package is used ^7^. The data can be accessed in R using data(jasa). For both Kaplan Meier survival analysis and quantile survival analysis, Level 3 RNASeqV2 Breast Cancer (BRCA) data was downloaded from the TCGA data portal ^4^. 553 patients that received radiotherapy and had survival information were used. Gene expression data for a specific biomarker gene was log2 transformed. The user has the choice to divide the data based on either the twenty-fifth percentile (set as default) or the fiftieth or the seventy-fifth, or an optimal cut point based on the martingale residuals. Similarly, for competing risk analysis, the user could choose the example data or upload their own. The example data consists of 35 patients with acute leukemia who underwent Hematopoietic stem cell transplantation (HSCT) affected by either AML or ALL ^6^ ( http://www.stat.unipg.it/luca/R).

The developmental repository is available at https://github.com/manalirupji/CASAS/.

Archived source code as at the time of publication: http://doi.org/10.5281/zenodo.832845 ^8^

License: CASAS is available under the GNU public license (GPL-3).
