# Supplementary material for: CASAS: Cancer Survival Analysis Suite, a web based application
Source: F1000Res. 2017 Jul 31;6:919. Originally published 2017 Jun 15. [Version 2] doi: 10.12688/f1000research.11830.2 (PMC5580407; doi:10.12688/f1000research.11830.2)
Supplement: Peer review history

# Peer review: Referee response for version 2

**DOI:** 10.5256/f1000research.13225.r25109

**Published:** 2017-08-23

**Authors:** Seon-Young Kim

**Affiliations:** 1 Genome Structure Research Center, Korea Research Institute of Bioscience and Biotechnology, Daejeon, Korea, South

The manuscript entitled ‘CASAS: Cancer Survival Analysis Suite, a web based application’ by Rupji *et al* describes a web server, a shiny R based tool for interactive survival analysis and visualization. They provide several kinds of survival analysis with detailed explanations and example data sets. The web application is easy to use and is likely to be useful for many researchers. I tested each of the examples and found that they work as described in the manuscript. If I add one comment, I found that the CASAS doesn’t support analysis of multiple genes at once, which is necessary when one wants to identify prognostic gene(s) from omics data. The authors may discuss this point and refer to an example of web servers that support the analysis of multiple genes (i.e http://appex.kr).

I have read this submission. I believe that I have an appropriate level of expertise to confirm that it is of an acceptable scientific standard.

## References

[1] KimSKHwan KimJYunSJKimWJKimSY: APPEX: analysis platform for the identification of prognostic gene expression signatures in cancer. *Bioinformatics*.2014;30(22) : 10.1093/bioinformatics/btu521 3284-6 10.1093/bioinformatics/btu521 25091586

# Response

**Published:** 2017-08-23

**Authors:** Manali Rupji

**Affiliations:** Emory University, USA

We thank the referee for their kind review. The CASAS tool supports several univariate, single gene analyses, the results of which are able to be summarized altogether in a table. The tool offers a comprehensive analyses with respect to each gene under study. Thus, the CASAS tool implements a forward selection algorithm for identifying statistically significant individual gene associations with clinical outcome.

# Peer review: Referee response for version 2

**DOI:** 10.5256/f1000research.13225.r24659

**Published:** 2017-08-01

**Authors:** Gang Han

**Affiliations:** 1 Department of Epidemiology and Biostatistics, Texas A&M University School of Public Health, College Station, TX, USA

The authors have answered all my comments. I have no more additional comments.

I have read this submission. I believe that I have an appropriate level of expertise to confirm that it is of an acceptable scientific standard.

# Response

**Published:** 2017-08-01

**Authors:** Manali Rupji

**Affiliations:** Emory University, USA

We thank the referee for taking the time to review our work, and thank them for the kind review.

# Peer review: Referee response for version 1

**DOI:** 10.5256/f1000research.12784.r24053

**Published:** 2017-07-06

**Authors:** Gang Han

**Affiliations:** 1 Department of Epidemiology and Biostatistics, Texas A&M University School of Public Health, College Station, TX, USA

Authors of this article attempted to facilitate the routine survival analysis for clinicians and general health science professionals by building a web based survival analysis software with underlying R programming. Because survival data has increased usage in clinical research, this work is useful to fill out the gap of survival analysis for researchers without training in statistics and programming. But given a number of limitations, its practical value is questionable. Due to the following multiple missing components, this article does not look complete. This reviewer suggests major revision to improve its usability:  

1. The first missing component is the test of model assumptions. The validity of the proportional hazard assumption was not described.
2. The second missing piece is the detailed description of required data format. For example, some practitioners may label event/censor as “1/0”, while others will write “not censored/censored.” What is the requirement for the data file header? For missing values, some may leave it blank, others may write “missing” or “unknown.” These things are all trivial for statisticians but useful for clinical users to know
  and bear in mind. The authors may add a separate section to emphasize the correct format.
3. The third missing piece is a comprehensive example. Although some figures were shown, potential users may look for a detailed example to follow. The authors had made the point that this software can perform certain survival analyses, but they failed to explain/illustrate how to perform these analyses. A major revision is necessary to answer the question of “how” in an example that looks similar to majority of the users’ data and analytical needs.

I have read this submission. I believe that I have an appropriate level of expertise to confirm that it is of an acceptable scientific standard, however I have significant reservations, as outlined above.

# Response

**Published:** 2017-07-20

**Authors:** Manali Rupji

**Affiliations:** Emory University, USA

We thank the reviewer for their thoughtful comments that have led to an improved paper.  Below, we respond individually to each comment.

*1. The first missing component is the test of model assumptions. The validity of the*

*proportional hazard assumption was not described.*

We appreciate this reviewer’s careful review of the tool and in bringing this point to our attention. A test for proportional hazards (PH) was included as part of the standard survival analysis option. We recognize that several methods exist for checking the proportional hazards assumption and results may vary depending on the method applied. In response to the reviewer’s comment, we have included under the documentation tab, “Read Me”, that the proportional hazards assumption is examined by applying the method of Schoenfeld residuals. If this method indicates a violation of the proportional hazards assumption as indicated by a significant test of PH (versus alternative of non-PH) based on such residuals, then other methods should be examined that are outside the scope of this tool since results may vary depending on the method implemented (Hiller *et al. *2015).    

Ref:

Hiller L, Marshall A, Dunn J.  Assessing violations of the proportional hazards assumption in cox regression:  does the chosen method matter?  Trials.  2015; 16(suppl 2): P134.

*2. The second missing piece is the detailed description of required data format.*

*For example, some practitioners may label event/censor as “1/0”, while others*

*will write “not censored/censored.” What is the requirement for the data file*

*header? For missing values, some may leave it blank, others may write*

*“missing” or “unknown.” These things are all trivial for statisticians but useful for*

*clinical users to know and bear in mind. The authors may add a separate section to emphasize the correct format*

We appreciate the reviewer’s point of clarity here and have addressed their recommendation of including a separate paragraph that highlights the required format. This new paragraph is listed under the, “Data and software availability” section in the paper.

*3. The third missing piece is a comprehensive example. Although some figures*

*were shown, potential users may look for a detailed example to follow. The*

*authors had made the point that this software can perform certain survival*

*analyses, but they failed to explain/illustrate how to perform these analyses. A*

*major revision is necessary to answer the question of “how” in an example that*

*looks similar to majority of the users’ data and analytical needs.*

In response to the reviewer’s comment, we have updated the one example initially used for illustrating the quantile survival analysis to also illustrate the following: standard survival analysis, a test of proportional hazards assumption, and selection of a cutpoint for a continuous variable. Additionally, we have updated the specific dataset for landmark analysis, to include the time corresponding to the time-dependent cohort variable. We have left the other previous examples provided in the initial submission to supplement the ‘how to’ of individual methods. While we recognize the importance of having a single example to streamline the methods of survival analysis, we also thought it important to emphasize that the methods are not dependent on each other and that they can be implemented as several stand-alone methods under one tool.
